# Predictors of mortality in connective tissue disease-associated pulmonary arterial hypertension: a cohort study

**DOI:** 10.1186/ar4051

**Published:** 2012-10-05

**Authors:** Gene-Siew Ngian, Wendy Stevens, David Prior, Eli Gabbay, Janet Roddy, Ai Tran, Robert Minson, Catherine Hill, Ken Chow, Joanne Sahhar, Susanna Proudman, Mandana Nikpour

**Affiliations:** 1 Department of Medicine, The University of Melbourne, Melbourne, VIC 3010, Australia; 2 Department of Rheumatology, St. Vincent's Hospital Melbourne, 41 Victoria Pde, Fitzroy, VIC 3065, Australia; 3 Department of Cardiology, St. Vincent's Hospital Melbourne, 41 Victoria Pde, Fitzroy, VIC 3065, Australia; 4 Advanced Lung Disease Unit, Royal Perth Hospital, GPO Box X2213, Perth, WA 6001, Australia; 5 Department of Rheumatology, Royal Perth Hospital, GPO Box X2213, Perth, WA 6001, Australia; 6 Department of Cardiovascular Medicine, Flinders Medical Centre, Flinders Drive, Bedford Park, SA 5042, Australia; 7 Department of Rheumatology, The Queen Elizabeth Hospital, 28 Woodville Rd., Woodville South, SA 5011, Australia; 8 Department of Rheumatology, Royal Adelaide Hospital, North Terrace, Adelaide, SA 5000, Australia; 9 Department of Rheumatology, Monash Medical Centre, 246 Clayton Rd., Clayton, VIC 3168, Australia

**Keywords:** Connective tissue disease, mortality, prognosis

## Abstract

**Introduction:**

Pulmonary arterial hypertension (PAH) is a major cause of mortality in connective tissue disease (CTD). We sought to quantify survival and determine factors predictive of mortality in a cohort of patients with CTD-associated PAH (CTD-PAH) in the current era of advanced PAH therapy.

**Methods:**

Patients with right heart catheter proven CTD-PAH were recruited from six specialised PAH treatment centres across Australia and followed prospectively. Using survival methods including Cox proportional hazards regression, we modelled for all-cause mortality. Independent variables included demographic, clinical and hemodynamic data.

**Results:**

Among 117 patients (104 (94.9%) with systemic sclerosis), during 2.6 ± 1.8 (mean ± SD) years of follow-up from PAH diagnosis, there were 32 (27.4%) deaths. One-, two- and three-year survivals were 94%, 89% and 73%, respectively. In multiple regression analysis, higher mean right atrial pressure (mRAP) at diagnosis (hazard ratio (HR) = 1.13, 95% CI: 1.04 to 1.24, *P *= 0.007), lower baseline six-minute walk distance (HR = 0.64, 95% CI: 0.43 to 0.97, *P *= 0.04), higher baseline World Health Organization functional class (HR = 3.42, 95% CI: 1.25 to 9.36, *P *= 0.04) and presence of a pericardial effusion (HR = 3.39, 95% CI: 1.07 to 10.68, *P *= 0.04) were predictive of mortality. Warfarin (HR = 0.20, 95% CI: 0.05 to 0.78, *P *= 0.02) and combination PAH therapy (HR = 0.20, 95% CI: 0.05 to 0.83, *P *= 0.03) were protective.

**Conclusions:**

In this cohort of CTD-PAH patients, three-year survival was 73%. Independent therapeutic predictors of survival included warfarin and combination PAH therapy. Our findings suggest that anticoagulation and combination PAH therapy may improve survival in CTD-PAH. This observation merits further evaluation in randomised controlled trials.

## Introduction

Pulmonary arterial hypertension (PAH) is a major cause of mortality in connective tissue disease (CTD), particularly in systemic sclerosis (SSc) [1]. Prior to the introduction of advanced PAH therapies, such as endothelin receptor antagonists (ERA), prostacyclin analogues and phosphodiesterase type-5 inhibitors (PDE5), treatment options for PAH were limited. Current therapies increase exercise tolerance and improve hemodynamic parameters [2]. A recent meta-analysis suggests that they also confer a survival benefit [3]. In one contemporary cohort of patients with SSc-associated PAH (SSc-PAH), survival was 81% at one year and 71% at two years, compared to 68% and 47% respectively, in a historical cohort (*P *= 0.016) [4]. Survival in CTD-associated PAH (CTD-PAH) is shorter than in idiopathic PAH (IPAH). This remains the case in the current treatment era, as demonstrated in a US registry of PAH patients wherein one-year survival was 86% among patients with CTD-PAH compared to 93% in patients with IPAH (*P *< 0.0001) [5].

Currently in Australia, prescription of specific PAH therapy is limited to government designated PAH treatment centres. All patients with right-heart catheter (RHC) proven PAH qualify for monotherapy with bosentan, ambrisentan, sildenafil or inhaled iloprost. For ongoing therapy, patients must demonstrate stability of both six-minute walk distance (6MWD) and echocardiographic parameters, although they are able to swap to an alternate agent if these criteria are not met. Combination PAH therapy is currently available at cost or on compassionate grounds. Sitaxentan was available in Australia until its worldwide withdrawal in early 2011. Intravenous prostacyclin analogues were not subsidised in the treatment of CTD-PAH in Australia at the time of this study.

In this study, our objective was to quantify survival and determine factors predictive of mortality among Australian patients with CTD-PAH, since the advent of specific PAH therapies.

## Materials and methods

All patients with CTD-PAH diagnosed on RHC [6] from November 2002 onwards were recruited (at the time of diagnosis of PAH) from six PAH treatment centres across Australia and followed prospectively, at three- to six-month intervals. Date of diagnosis of PAH was defined as the date of the RHC.

Clinical and hemodynamic variables were recorded during follow-up. Data were censored at 31 December 2009 for analysis. Ethics approval was obtained from the human research ethics committees of St. Vincent's Hospital Melbourne, Southern Health, Royal Adelaide Hospital, Royal Perth Hospital and Central Northern Adelaide Health Service. Patients in this study provided informed consent.

### Demographic and disease-related variables

All patients had SSc or another underlying CTD, namely rheumatoid arthritis (RA), systemic lupus erythematosus (SLE) or mixed connective tissue disease (MCTD). Definitions were based on the American College of Rheumatology diagnostic criteria for SSc [7], RA [8] and SLE [9] and the Alarcon-Segovia diagnostic criteria for MCTD [10]. Patients were defined as having limited or diffuse SSc according to the classification criteria of LeRoy *et al. *[11]. World Health Organization (WHO, Geneva, Switzerland) functional class (FC) [12] and 6MWD [13] at PAH diagnosis were recorded along with echocardiographic and RHC parameters. The presence of pericardial effusion on echocardiography was documented. We excluded patients with significant interstitial lung disease (ILD) defined based on extensive disease (> 20% lung involvement) on high-resolution CT lung (HRCT), or evidence of fibrosis on HRCT together with forced vital capacity (FVC) < 70% predicted and/or an FVC to diffusing capacity of carbon monoxide (DLCO) ratio < 1.6 in whom PAH was deemed secondary to lung disease [14]. Other covariates included autoantibody profile, anti-phospholipid antibody status (positive if either anti-cardiolipin or anti-beta2 glycoprotein IgG or IgM greater than upper limit of normal for assay) and the PAH study centre from which the patient was recruited.

### Outcome variable

The outcome variable was all-cause mortality. The date of death was recorded. Where data were available, the exact causes of death were also recorded and verified through chart review. The status (alive or dead) of patients at the time of censoring was confirmed by checking with the treating physician and through chart review. There were no losses to follow-up.

### Treatment related variables

Specific PAH therapies, prescribed at physician discretion following RHC-confirmed diagnosis of PAH, were recorded at each visit. This included 'combination therapy', which was defined as treatment with more than one agent from any of the three classes (ERA, PDE5 inhibitors, prostacyclin analogues) at the same time. Indications for substitution or addition of a second agent were documented. We also recorded warfarin therapy of at least six months' duration, following PAH diagnosis. Indications and complications of warfarin therapy, and target INR were also recorded. In those who were not anticoagulated, we recorded contraindications for warfarin therapy as documented by the treating physician.

### Statistical analysis

Patient characteristics at baseline are reported as mean ± standard deviation (SD) for continuous variables and proportions (percentages) for categorical variables. Differences in baseline hemodynamics between SSc patients and other CTD patients, and patients on monotherapy vs combination therapy were compared using the Student's *t*-test. Kaplan-Meier (K-M) curves were used to estimate survival in all patients and also in SSc patients compared with those who had other CTDs. Log-rank and Wilcoxon tests were used to determine univariate predictors of survival. After testing to ensure proportionality of hazard, Cox proportional hazards regression analyses were used to determine univariate and multivariable predictors of survival. These results were reported as hazard ratios (HR) with accompanying 95% confidence intervals (95% CI). Two-tailed *P-*values ≤ 0.05 were considered statistically significant.

All statistical analyses were performed using STATA 11.0 (Statacorp, College Station, TX, USA).

## Results

A total of 117 patients with incident CTD-PAH were recruited. Patient characteristics and baseline hemodynamics are summarised in Table 1. During a mean ± SD follow-up from PAH diagnosis of 2.6 ± 1.8 years, there were 32 (27.4%) deaths. All but three deaths were primarily due to PAH. In the remaining three deaths due to malignancy, PAH was a major contributor to death.

**Table 1:** Patient characteristics at baseline

Characteristic	Mean + SD or n(%)
Total number of patients	117
Female	105 (89.7%)
Age at PAH diagnosis*, years	61.5 ± 11.4
Disease duration at PAH diagnosis*, years	11.7 ± 11.3
Race	
Caucasian	101 (86.3%)
Asian	10 (8.5%)
Aboriginal/Torres Strait Islander	3 (2.6%)
Hispanic	1 (0.9%)
Other	2 (1.7%)
Underlying CTD	
Limited SSc	79 (72.5%)
Diffuse SSc	25 (21.4%)
MCTD	5 (4.5%)
SLE	3 (2.8%)
RA	3 (2.8%)
Undifferentiated CTD	2 (1.8%)
Anti-centromere antibody positive	56 (47.9%)
Anti-Scl-70 antibody positive	9 (7.7%)
Anti-phospholipid antibodies	27 (23.1%)
WHO functional class	
Class I	9 (7.7%)
Class II	14 (12.0%)
Class III	88 (75.2%)
Class IV	6 (5.1%)
Baseline 6MWD, m	325 ± 127
Baseline mRAP, mm Hg	6.9 ± 4.2
Baseline mPAP, mm Hg	35.9 ± 12.4
Baseline PCWP, mmHg	9.7 ± 3.9
Baseline CI, L/min/m^2^	2.6 ± 0.8
Baseline PVR, Wood units	6.9 ± 6.1
Pericardial effusion	14 (12.0%)
Warfarin therapy	36 (30.8%)
Pulmonary vasodilator therapy	
Monotherapy	70 (59.8%)
Sequential monotherapy	12 (10.3%)
Combination therapy	34 (29.0%)

* date of PAH diagnosis is the date of right heart catheterisation

6MWD, six minute walk distance; CI, cardiac index; CTD, connective tissue disease; MCTD, mixed connective tissue disease; mPAP, mean pulmonary arterial pressure; mRAP, mean right atrial pressure; PAH, pulmonary arterial hypertension; PVR, pulmonary vascular resistance; RA, rheumatoid arthritis; SD, standard deviation; SLE, systemic lupus erythematosus; SSc, systemic sclerosis; WHO, World Health Organization

### Specific PAH therapy

All patients received specific PAH therapy. Seventy patients (59.8%) received monotherapy, 12 patients (10.3%) sequential monotherapy and 34 patients (29.0%) combination therapy (Table 1). The main reasons for sequential and combination therapy were failure of initial PAH therapy or drug-related adverse effects significant enough to warrant a change in therapy.

Bosentan was the most commonly prescribed medication, used in 103 patients (88.0%). Sildenafil was the next most common medication used in 38 patients (32.5%), followed by sitaxentan in 18 (15.4%) and inhaled iloprost in 15 (13.4%). The most common combination therapies were bosentan and sildenafil (21 patients), followed by bosentan, sildenafil and inhaled iloprost (6 patients).

### Comparisons of baseline clinical and hemodynamic data

Mean ± SD mPAP at baseline was lower in patients with SSc than in patients with other CTDs (34.3 ± 11.7 mmHg vs 49.5 ± 10.3 mmHg, *P *< 0.001). SSc patients were also older at PAH diagnosis (62.6 ± 10.3 years vs 54.0 ± 17.1 years, *P *= 0.01) and had higher baseline 6MWD (334.6 ± 125.2 m vs 235.0 ± 110.3 m, *P *= 0.02) than patients with other CTDs.

Among all patients in the study, at baseline, those who subsequently received combination therapy had higher mPAP than those who received monotherapy or sequential monotherapy (40.0 ± 11.6 mmHg vs 34.0 ± 12.4 mmHg, *P *= 0.02). There were no differences in age at PAH diagnosis (59.6 ± 11.5 vs 62.5 ± 11.4 years, *P *= 0.21) or baseline 6MWD (303.3 ± 114.5 m vs 333.8 ± 131.5 m, *P *= 0.27) among the combination vs monotherapy or sequential monotherapy treatment groups.

### Survival and factors predictive of mortality

**Figure 1:**
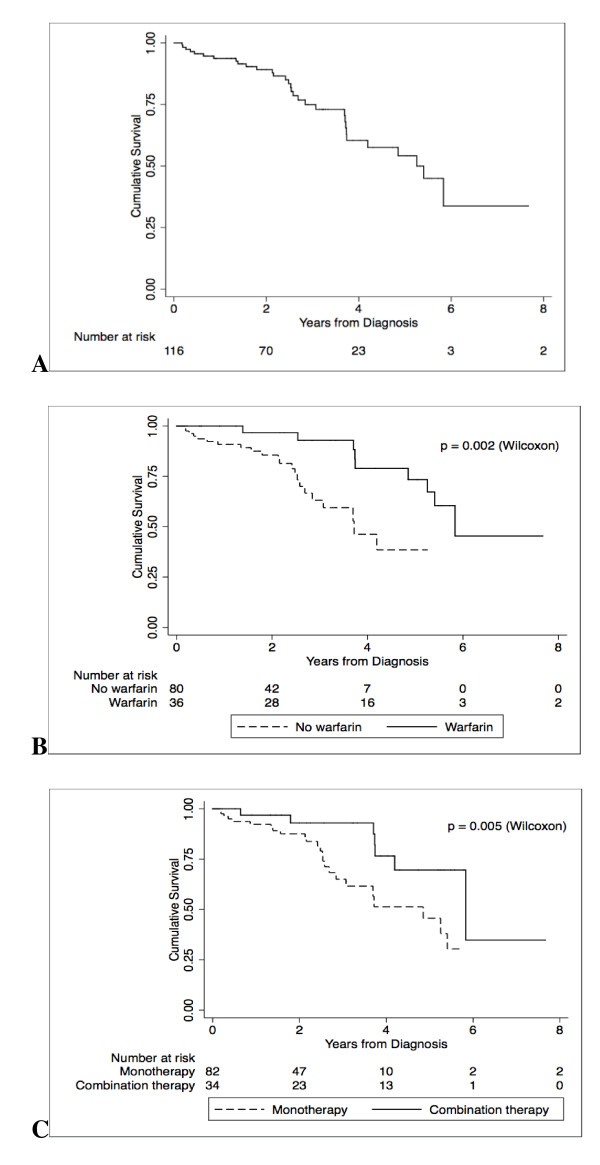
**Survival from PAH diagnosis - (A) all patients and grouped by (B) warfarin therapy and (C) combination PAH therapy**. PAH, pulmonary arterial hypertension.

Twenty-seven of the 32 (84.4%) deaths occurred in patients with SSc. Overall, one-year survival was 94%, two-year survival was 89% and three-year survival was 73% (Figure 1A). Median survival was approximately five years.

In univariate analysis (Table 2), factors associated with mortality were male sex, higher baseline mRAP on RHC, higher baseline WHO FC, lower baseline 6MWD, pericardial effusion, absence of warfarin therapy (Figure 1B) and lack of combination therapy (Figure 1C).

**Table 2:** Predictors of mortality in CTD-PAH on univariate analysis

Variable	Unadjusted HR (95% CI)	*P*
Male sex	3.42 (1.27 to 9.22)	0.02
Age at PAH diagnosis, years	1.01 (0.98 to 1.04)	0.72
Underlying CTD (SSc)	1.27 (0.44 to 3.66)	0.66
WHO FC at baseline	3.64 (1.32 to 10.01)	0.01
Baseline 6MWD*	0.70 (0.52 to 0.95)	0.02
Pericardial effusion	2.83 (1.12 to 7.12)	0.03
mPAP at baseline, mmHg	1.01 (0.98 to 1.05)	0.42
mRAP at baseline, mmHg	1.11 (1.02 to 1.19)	0.01
Warfarin therapy	0.26 (0.11 to 0.66)	0.004
Combination therapy	0.38 (0.16 to 0.92)	0.03

*hazard ratio relates to each 100 metres increase in 6MWD

6MWD, six minute walk distance; CTD, connective tissue disease; HR, hazard ratio; mPAP, mean pulmonary artery pressure; mRAP, mean right atrial pressure; PAH, pulmonary arterial hypertension; SSc, systemic sclerosis; WHO FC, World Health Organization Functional Class

**Figure 2:**
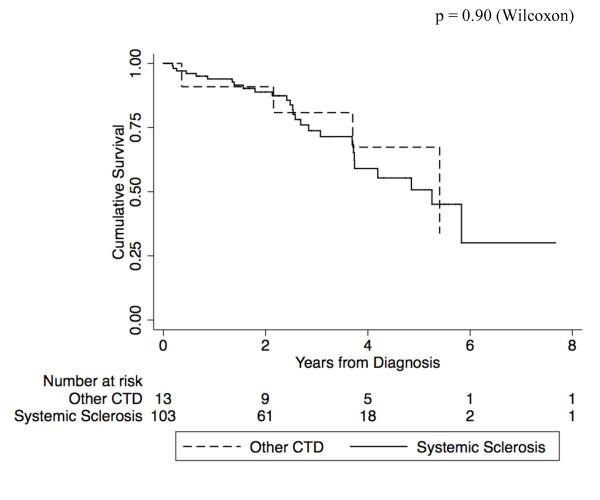
**Survival from PAH diagnosis of patients with SSc compared with those with other CTDs**. PAH, pulmonary arterial hypertension; SSc, systemic sclerosis; CTD, connective tissue disease.

Cox regression analysis was performed to make adjustments for the effect of multiple covariates. In regression analysis, we took into consideration a desired ratio of independent-to-outcome variables of at most one to five, in order to ensure model stability (Table 3) [15]. Higher mRAP at diagnosis (HR = 1.13, 95% CI: 1.04 to 1.24, *P *= 0.007), lower baseline 6MWD (HR = 0.64, 95% CI: 0.43 to 0.97, *P *= 0.04), higher baseline WHO FC (HR = 3.42, 95% CI: 1.25 to 9.36, *P *= 0.04) and pericardial effusion (HR = 3.39, 95% CI: 1.07 to 10.68, *P *= 0.04) were predictive of mortality. Warfarin (HR = 0.20, 95% CI: 0.05 to 0.78, *P *= 0.02) and combination pulmonary vasodilator therapy (HR = 0.20, 95% CI: 0.05 to 0.83, *P *= 0.03) were protective. Male sex was not independently associated with mortality and, therefore, was removed from the final model. Overall, survival in patients with SSc (*n *= 104) was not significantly different compared to those with other CTDs (*n *= 13) (Figure 2). In further regression analyses of the SSc patients only, treatment with warfarin (HR = 0.33, 95% CI: 0.11 to 0.96, *P *= 0.04) and combination therapy (HR = 0.55, 95% CI: 0.33 to 0.92, *P *= 0.02) remained protective.

**Table 3:** Independent predictors of mortality in CTD-PAH, determined using multivariable proportional hazards regression analysis

Variable	Adjusted HR (95% CI)	*P*
WHO FC at baseline	3.42 (1.25 to 9.36)	0.04
mRAP at baseline, mmHg	1.13 (1.04 to 1.24)	0.007
Baseline 6MWD*, m	0.64 (0.43 to 0.97)	0.04
Pericardial effusion	3.39 (1.07 to 10.68)	0.04
Warfarin therapy	0.20 (0.05 to 0.78)	0.02
Combination therapy	0.20 (0.05 to 0.83)	0.03

*hazard ratio relates to each 100 metres increase in 6MWD

6MWD, six minute walk distance; 95% CI, 95% confidence interval; HR, hazard ratio; mRAP, mean right atrial pressure; WHO FC, World Health Organization functional class

### Warfarin therapy

Twenty-eight of 36 (77.8%) patients on warfarin had SSc, 2 had MCTD, 2 SLE and 3 RA, and 1 had an undifferentiated CTD. Ten of 36 patients on warfarin died during follow-up. Among these, 7 had SSc, 1 had SLE, 1 MCTD and 1 RA. The main indication for anticoagulation in all 36 patients was PAH. However, three patients also had atrial fibrillation and three patients had a history of deep vein thrombosis (DVT). Of note, in the three patients with the history of DVT, PAH was deemed related to the CTD (WHO diagnostic group 1) [16] and not pulmonary thromboembolic disease. The target International Normalised Ratio (INR) in all patients was 2.0 to 3.0.

Univariate comparisons of characteristics of patients treated with warfarin, compared to those who were not treated with warfarin are summarised in Table 4. Patients on warfarin were significantly more likely to have a pericardial effusion (42.0% vs 33.4%, *P *= 0.001) and a higher baseline mPAP (42.0 vs 33.4 mm Hg, *P *= 0.001) than those not receiving warfarin. We found that there was no significant difference in baseline mRAP or baseline 6MWD between patients who were and those who were not anticoagulated (Table 4).

**Table 4:** Univariate comparison of characteristics of patients who were anticoagulated and those who were not anticoagulated

Characteristic	Anticoagulated (*n *= 36)	Not anticoagulated (*n *= 81)	*P*
		
	Mean ± SD or n (%)	Mean ± SD or n (%)	
Female	34 (94.4%)	71(87.6%)	0.26
Age at CTD diagnosis, years	48.5 ± 14.3	51.1 ± 14.7	0.46
Age at PAH diagnosis*, years	59.7 ± 13.1	62.5 ± 10.6	0.23
Anti-phospholipid antibodies	8 (22.1%)	19 (23.5%)	0.74
Contraindication to anticoagulation	0 (0%)	18 (22.2%)	0.002
WHO functional class			
Class I	0 (0%)	9 (11.1%)	
Class II	3 (8.3%)	11 (13.6%)	0.09
Class III	32 (88.9%)	56 (69.1%)	
Class IV	1 (2.8%)	5 (6.2%)	
Baseline 6MWD, m	286.3 ± 116.4	339.5 ± 128.4	0.06
Baseline mRAP, mmHg	7.1 ± 4.3	6.9 ± 4.1	0.85
Baseline mPAP, mmHg	42.0 ± 11.5	33.4 ± 12.0	0.001
Pericardial effusion	9 (25.0%)	5 (6.2%)	0.004
Pulmonary vasodilator therapy			
Monotherapy	13 (36.1%)	57 (71.3%)	
Sequential monotherapy	2 (5.6%)	10 (12.5%)	< 0.0001
Combination therapy	21 (58.3%)	13 (16.3%)	

* date of PAH diagnosis is the date of right heart catheterisation

6MWD, six minute walk distance; CTD, connective tissue disease; ILD, interstitial lung disease; mPAP, mean pulmonary arterial pressure; mRAP, mean right atrial pressure; PAH, pulmonary arterial hypertension; SD, standard deviation; SSc, systemic sclerosis; WHO, World Health Organization

Among patients who were not treated with warfarin, a contraindication to anticoagulation was present in only 18 of 81 (22.2%) patients, and in the remainder the decision to not treat with warfarin was made at physician discretion. The most common contraindication in 16 patients was a history of gastrointestinal bleeding, with two other patients having a history of recurrent falls. On follow-up, only one patient with SSc, who was on warfarin for the treatment of PAH, had a complication of GI bleeding.

## Discussion

In this cohort study of patients with CTD-PAH confirmed by RHC, one- two- and three-year survivals were 94%, 89% and 73%, respectively. We found that independent predictors of mortality were more severe PAH at diagnosis, manifested by higher baseline mRAP, lower baseline 6MWD, higher baseline WHO FC and the presence of pericardial effusion. After adjustment for these covariates, anticoagulation and combination PAH therapy increased survival.

Our one-year survival of 94% is higher than the one-year survival of 86% reported by Chung *et al. *[5] in a cohort of CTD-PAH patients from a multi-centre US-based registry. Bearing in mind that the majority (95%) of patients in our study had SSc, our survival rates are also higher than those reported by Condliffe *et al. *[17] from the UK pulmonary hypertension service, where one-year survival was 78% and three-year survival was 47% in SSc-PAH, while one-year survival was 78% and three-year survival was 74% in SLE-associated PAH. There are several possible explanations for the differences in survival rates of our patients compared with other cohorts. These include differences in hemodynamic profile at PAH diagnosis and variations in the approach to treatment, with a significant proportion of our patients receiving combination therapy. In addition, it is possible that other cohorts may contain patients with overall more severe CTD, who are more likely to succumb to their disease.

Whilst elevated mRAP was associated with mortality in our study, elevated mPAP was not significantly associated with mortality in either univariate or multivariable analysis. This apparent discrepancy between mPAP and mRAP may be due to a proportion of patients having myocardial disease due to SSc, thereby compromising the ability of the right ventricle to generate an elevated mPAP. On the other hand, mRAP better reflects right ventricular dysfunction and, therefore, continues to increase with worsening disease. These relationships were borne out in a systematic review of predictors of survival in IPAH which found that whilst 10 publications evaluating this issue supported the association between increased mPAP and decreased survival, 19 did not [18]. In the same systematic review, mRAP was the variable most commonly associated with mortality, with an independent predictive value for mortality reported in 17 of 28 studies.

6MWD is predictive of mortality in IPAH [19], and has been used as a primary endpoint in clinical trials of PAH therapy [20-24]. As 6MWD may be reduced due to impaired mobility and joint or muscle pain, especially in patients with CTD, some experts have considered it to be a less useful measure of exercise capacity in those with CTD-PAH than in those with IPAH [25]. Our findings suggest that, despite these limitations in certain individuals, 6MWD may still be a useful predictor of mortality in CTD-PAH.

In our study, higher baseline WHO FC was also independently predictive of mortality. The prognostic value of WHO or New York Heart Association FC is supported by multiple studies in PAH, several of which have included patients with CTD-PAH [18]. Lack of improvement in FC after specific PAH therapy has also been suggested as an indication to escalate monotherapy to combination therapy [2]. However, WHO FC is a subjective measure and does not always correlate with other markers of PAH severity, such as mRAP and 6MWD [26].

In IPAH, the presence of pericardial effusion has been reported to be predictive of mortality in two retrospective studies, and the severity of pericardial effusion has been predictive of mortality in five retrospective studies [18]. In our study, the presence of a pericardial effusion was predictive of mortality in CTD-PAH. This is consistent with the findings of Fisher *et al. *[27]. Although the physiology of the accumulation of fluid in the pericardial space is incompletely understood, pericardial effusion in this context is likely due to increased venous pressure and right heart failure [28]. Importantly, in our patients, there were no echocardiographic or other clinical features of serositis or pericarditis to indicate active inflammatory disease as the cause for pericardial effusion.

In multiple regression analysis, after adjustment for covariates, including use of specific PAH therapies, we found that anticoagulation with warfarin conferred a substantial survival benefit in CTD-PAH, which has not previously been reported. Correlates of warfarin therapy included more severe PAH, with anticoagulated patients more likely to have a higher mPAP and a pericardial effusion. This is consistent with an international survey of PAH experts, which found that clinicians were more likely to use warfarin in more severe CTD-PAH [29]. This survey also revealed variation in physician beliefs and prescribing habits in relation to warfarin in CTD-PAH. Whilst it is accepted that thrombotic arteriopathy plays a role in the pathophysiology of PAH [30], the evidence for a survival benefit with anticoagulation remains limited. A survival benefit was suggested by a systematic review of warfarin therapy in IPAH in which five of seven retrospective analyses reported a survival benefit with warfarin use [31]; no CTD-PAH patients were included in this analysis. A recent observational study of 275 patients with SSc-PAH showed a low probability of survival with warfarin; however, in this study, over a third of the patients on warfarin were not receiving specific PAH therapies [32].

In our study, the most common documented contraindication to anticoagulation in 16 patients was a history of gastrointestinal bleeding. This is a major consideration in patients with SSc-PAH as the prevalence of gastrointestinal vascular ectasia, a possible source of blood loss, is reported to be as high as 5.7% [33].

It is possible that our finding of a survival benefit with anticoagulation was confounded by sicker and frailer patients with severe multi-organ CTD being less likely to receive warfarin therapy. While data on PAH severity and other SSc manifestations were collected, we did not systematically collect data on non-SSc-related comorbidities which may have impacted the patients' overall health status and suitability for anticoagulation. Despite our efforts to adjust for all clinically important covariates, our observational study cannot fully overcome the effect of all potential confounders. Evaluation of the therapeutic efficacy of anticoagulation in CTD-PAH warrants a more definitive study, with random assignment of treatment in patients with similar baseline characteristics.

Combination specific PAH therapy in CTD-PAH targets more than one of the multiple biologic pathways involved in disease pathogenesis [2]. Although a number of small, short-term trials of dual agent therapy have been conducted, results have been disparate and there are no data available beyond 12 weeks of therapy [2]. In a cohort of 112 patients with PAH, among whom 40 had CTD (29 had SSc), Keogh *et al. *recently reported survival of 72% at 12 months and 48% at 24 months in patients on dual specific PAH therapy [34].

In this study, we have sought to determine survival and predictors of mortality in a cohort of CTD-PAH patients sourced from multiple centres, typical of those encountered in clinical practice. Although we included all CTD-PAH patients at each site, there was potential for selection bias in that the sites included in our study were large academic centres. However, in practice, as prescription of CTD-PAH therapy in Australia is limited to large government designated centres, patients with all grades of PAH severity are referred to these sites for treatment.

## Conclusions

Overall, the findings of this study suggest that treatment with warfarin in addition to combination specific PAH therapy may improve survival in CTD-PAH. The efficacy of anticoagulation and combination therapy in CTD-PAH merits further evaluation.

## Abbreviations

6MWD: six minute walk distance; 95% CI: 95% confidence interval; CI: cardiac index; CTD: connective tissue disease; CTD-PAH: connective tissue disease-associated pulmonary arterial hypertension; DLCO: diffusing capacity of carbon monoxide; DVT: deep vein thrombosis; ERA: endothelin receptor antagonists; FVC: forced vital capacity; GI: gastrointestinal; HR: hazard ratio; HRCT: high-resolution computed tomography; ILD: interstitial lung disease; INR: International Normalised Ratio; IPAH: idiopathic pulmonary arterial hypertension; K-M curves: Kaplan-Meier curves; MCTD: mixed connective tissue disease; mPAP: mean pulmonary arterial pressure; mRAP: mean right atrial pressure; PAH: pulmonary arterial hypertension; PDE5: phosphodiesterase type-5 inhibitors; PVR: pulmonary vascular resistance; RA: rheumatoid arthritis; RHC: right-heart catheter; SD: standard deviation; SLE: systemic lupus erythematosus; SSc: systemic sclerosis; WHO FC: World Health Organization Functional Class.

## Competing interests

Authors WS, EG and SP have received consultancies and speaking fees from Actelion Australia, GalxoSmithKline and Pfizer (amounts less than $10,000). Other authors have no disclosures.

## Authors' contributions

GSN and MN contributed to study design, data collection and analysis, interpretation of findings and preparation of the manuscript. WS and SP contributed to study design, data collection, interpretation of findings and preparation of the manuscript. DP, EG, JR, AT, RM, CH, KC and JS contributed to data collection, interpretation of findings and preparation of the manuscript. All authors have approved the manuscript for publication.
